# Decentralized community-based hub-intermediary-spoke model for rapid cardiac ultrasound triage for early heart failure detection: findings from the Heart2Miss initiative

**DOI:** 10.1093/ehjdh/ztag115

**Published:** 2026-08-10

**Authors:** Diana Hui Ping Foo, John Jui Ping Yeo, Yenny Yen Yen Yeo, Stephanie Tayas Bumphray, Rose Hui Chin Jong, Liana Lantong Sumbu, Claudia Lennya Jana, Jennett Michael, Maziah Ishak, Peter Jerampang, Maila Mustapha, Sally Suriani Ahip, Lenny Hamden, Macnicholson Igo, Mohammad Nor Azlan Sulaiman, Jawing Chunggat, Francesca Gelang Chong, Deborah Berenai Enggong, Yeequant Chung, Daniel Ishak, Angela Melinda Anthony Jalin, Sonesh Kalra, Alan Yean Yip Fong

**Affiliations:** Clinical Research Centre, Sarawak General Hospital, Kuching 93586, Malaysia; Clinical Research Centre, Sarawak General Hospital, Kuching 93586, Malaysia; Clinical Research Centre, Sarawak General Hospital, Kuching 93586, Malaysia; Clinical Research Centre, Sarawak General Hospital, Kuching 93586, Malaysia; Clinical Research Centre, Sarawak General Hospital, Kuching 93586, Malaysia; Clinical Research Centre, Sarawak General Hospital, Kuching 93586, Malaysia; Clinical Research Centre, Sarawak General Hospital, Kuching 93586, Malaysia; Klinik Kesihatan Kota Sentosa, Kuching 93250, Malaysia; Klinik Kesihatan Petra Jaya, Kuching 93050, Malaysia; Klinik Kesihatan Tanah Puteh, Kuching 93450, Malaysia; Klinik Kesihatan Jalan Masjid, Kuching 93000, Malaysia; Klinik Kesihatan Kota Samarahan, Kota Samarahan 94300, Malaysia; Klinik Kesihatan Batu Kawa, Kuching 93250, Malaysia; Clinical Research Centre, Sarawak General Hospital, Kuching 93586, Malaysia; Clinical Research Centre, Sarawak General Hospital, Kuching 93586, Malaysia; Clinical Research Centre, Sarawak General Hospital, Kuching 93586, Malaysia; Clinical Research Centre, Sarawak General Hospital, Kuching 93586, Malaysia; Clinical Research Centre, Sarawak General Hospital, Kuching 93586, Malaysia; Us2.ai, Singapore, Singapore; Us2.ai, Singapore, Singapore; Department of Neurosurgery, Neuroscience Institute, Penn State Hershey Medical Centre, Hershey 17033, USA; AstraZeneca (Malaysia) Sdn Bhd, Selangor, Malaysia; Clinical Research Centre, Sarawak General Hospital, Kuching 93586, Malaysia; Department of Cardiology, Sarawak Heart Centre, Kota Samarahan 94300, Malaysia

**Keywords:** Type 2 diabetes, Heart failure, Artificial intelligence, Point-of-care ultrasound, Decentralized care

## Abstract

**Aims:**

To evaluate the feasibility and system-level performance of Heart2Miss, a decentralized community-based triage model deploying AI-powered point-of-care ultrasound (AI-POCUS) via a hub-intermediary-spoke approach in diabetes primary care for early heart-failure (HF) detection.

**Methods and results:**

In this prospective study, 1000 adults with diabetes and no known HF were screened over seven months across six primary care clinics (spokes); 985 with complete data were analysed. Novice biomedical and bioscience graduates underwent 4-week training to perform focused three-view handheld AI-POCUS. Images were AI-analysed and verified through the hub–intermediary–spoke pathway. The primary outcome was detection of previously undiagnosed HF. Secondary outcomes included reduction in tertiary-centre burden through the hub–intermediary–spoke pathway and novice sonographer performance. 11.1% (*n* = 109) had Stage B (pre-HF) and 1.0% (*n* = 10) Stage C HF (symptomatic HF). Rapid triage ruled out abnormality in 77.3% at the spoke and a further 12.6% after intermediary TTE confirmation, reducing tertiary diagnostic burden by 89.9%. Only 1.0% required tertiary referral. Regarding novice performance, >90% analysable scans were achieved for left-ventricular parameters and >85% for left-atrial volume. After 400 scans, scan time fell from 11.0 ± 5.3 min to 8.3 ± 4.4 min (Δ 2.31 min, 95% CI 1.52–3.11; *P* < 0.001), and complete three-view capture improved from 88.0% to 92.2% (*P* = 0.035).

**Conclusion:**

This decentralized hub–intermediary–spoke model combining AI-POCUS, telehealth verification, and a task-shifted bioscience workforce enabled early HF detection while substantially reducing specialist workload, supporting digital health-enabled workforce innovation and pathway redesign in resource-constrained settings.

## Graphical Abstract

**Graphical Abstract:**
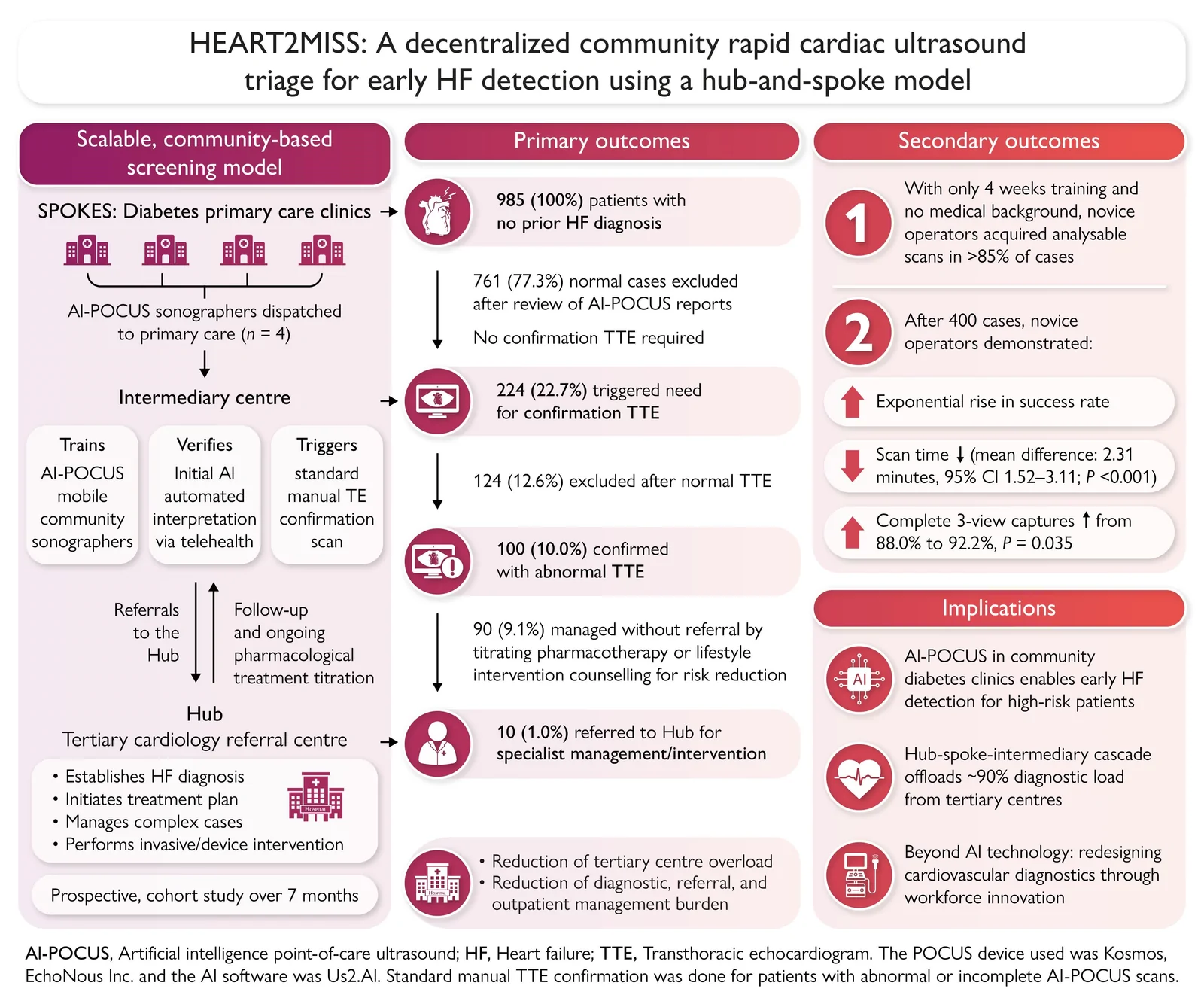

## Introduction

Heart failure (HF) remains a major public health challenge affecting an estimated 64 million people worldwide, and is the leading cause of hospitalization among individuals aged over 65 years^1,2^ In many patients, structural heart changes and elevated cardiac biomarkers precede the onset of symptoms by years. Yet more than 40% of patients report HF symptoms for up to 5 years before receiving a diagnosis, missing a critical window for early intervention and improved outcomes.^3^ With the global prevalence of diabetes, particularly type 2 diabetes mellitus (T2DM), increasing by over 30% in the past decade, the burden of HF is expected to rise further, posing significant challenges to healthcare systems worldwide.^4,5^

Individuals with T2DM face particularly high cardiovascular (CV) risk, with cardiovascular disease (CVD) accounting for up to 80% of excess mortality and HF incidence approximately 2.5 times higher compared with non-diabetic controls.^6^ Among hospitalized patients with diabetes, HF prevalence ranges from 12% to 22%.^6^ The combination of T2DM and chronic kidney disease (CKD) doubles the risk of one-year mortality or HF-related hospitalization in patients with both reduced (HFrEF) and preserved (HFpEF) ejection fraction.^7^ Despite these risks, up to 50% of patients with HF, including those with T2DM, remain undiagnosed in the primary care settings,^6^ largely due to lack of access to advanced cardiac diagnostics. Access to echocardiography (ECHO), the gold standard for HF diagnosis, is often restricted by equipment costs, workforce shortages, and the need for specialist interpretation. While recent evidence suggests that proactive diagnostic strategies combining symptom and risk factor questionnaires can significantly increase CVD and HF detection in high-risk primary care populations,^8^ many resource-constrained regions still lack the tools, training, and capacity for comprehensive CV diagnostics.^9^

Point-of-care ultrasound (POCUS) has emerged as a promising technology for decentralized diagnostic assessment, offering portability, rapid image acquisition, and the ability to complement physical examination at the bedside or in the community.^10^ Originally applied in emergency and acute care settings, POCUS has demonstrated utility in the rapid evaluation of cardiac wall motion abnormalities and other pathologies using portable devices since the 1980s.^11^ More recently, its use has expanded into primary care, often led by trained physicians and nurses. However, successful applications have also been demonstrated among individuals without formal clinical training, such as medical students.^12^ Advancements in artificial intelligence (AI) have further enhanced the accessibility and utility of POCUS by automating image analysis and improving diagnostic accuracy. AI-powered POCUS (AI-POCUS) has shown particular promise in the automated measurement of left ventricular ejection fraction (LVEF), inferior vena cava collapsibility, left ventricular outflow tract velocity time integral, and the detection of B-lines in lung ultrasound, among other applications.^13^ While AI-POCUS has primarily been studied in acute care environments, emerging data suggest its potential to facilitate screening and triage in non-acute and resource-limited settings.

However, technology alone is insufficient without a service model that embeds it into clinical pathways. The Heart2Miss initiative was launched as a community-based programme using a decentralized hub–intermediary–spoke approach that combines AI-POCUS with task-shifting to trained biomedical and bioscience graduates to support rapid HF triage in high-risk individuals with T2DM in primary care clinics. This study evaluated the feasibility and system-level performance of a decentralized community-based hub–intermediary–spoke model for early HF detection in high-risk individuals with T2DM receiving routine care in primary care settings.

## Methods

### Study design and setting

**Figure 1:**
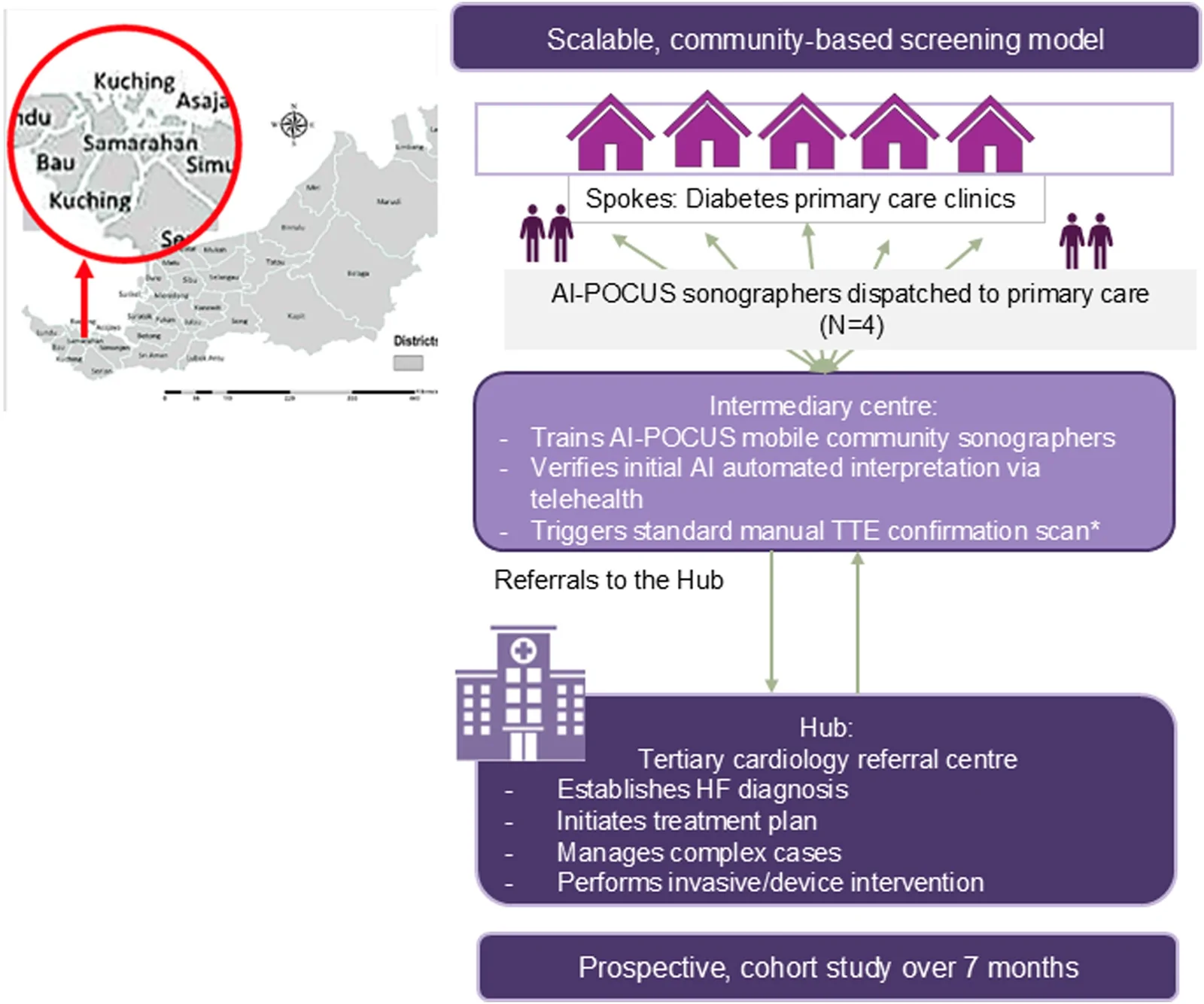
Scalable, community-based hub–spoke–intermediary model for early HF screening. The inset map highlights the Kuching and Samarahan districts of Sarawak, Malaysia. AI-POCUS, artificial intelligence-powered point-of-care ultrasound; HF, heart failure; TTE, transthoracic echocardiogram.

The Heart2Miss initiative was a prospective, decentralized, community-based study that utilized AI-POCUS triage within diabetes primary-care clinics through a hub– intermediary–spoke model (*Figure 1*). Six diabetes primary-care clinics in the Kuching and Samarahan districts of Sarawak, a state in Malaysia, served as spokes. Kuching District, which houses the state's administrative centre, Kuching city, covers 1 868.83 km^2^ and has an estimated population of 514 658, whereas the neighbouring Samarahan District spans 407.08 km^2^ with approximately 136 500 inhabitants.^14,15^

### Hub-intermediary-spoke model and functional roles

The Heart2Miss hub-intermediary-spoke model was designed to extend ECHO diagnostics into the community while optimizing specialist resources. The model comprises three functional tiers, i.e. the hub, intermediary and spokes.

The hub is a tertiary cardiology referral facility responsible for advanced diagnostics, confirmatory HF diagnosis, and specialist-level intervention and management for patients requiring specialist care. In this study, the hub was the Sarawak Heart Centre in Kuching city. The intermediary is the operational core of the network responsible for delivering structured training to novice biomedical and bioscience graduates to serve as mobile community AI-POCUS sonographers. Additionally, it functions as a core ECHO laboratory to verify all AI-automated analyses from the community AI-POCUS quick scans via telehealth, performs full confirmatory transthoracic echocardiography (TTE), and facilitates referrals to the hub as required. In this proof-of-concept model, the Sarawak General Hospital Clinical Research Centre served as the intermediary centre. The spokes were six diabetes primary care clinics in the public healthcare setting across Kuching and Samarahan districts. The trained mobile community sonographers were deployed to perform AI-POCUS quick scans as part of the routine T2DM investigations during follow-up visits.

### Study population

Adults (≥18 years) with T2DM, no prior HF diagnosis, and active follow-up in diabetes primary care clinics were prospectively screened. Inclusion criteria were the ability to provide informed consent and willingness to undergo AI-POCUS scanning as part of the study protocol. Exclusion criteria included known HF diagnosis (Stage C or D), haemodynamic instability, inability to tolerate scanning, or any condition precluding acquisition of adequate images. Consecutive eligible patients were invited to participate, and written informed consent was obtained before enrolment. Baseline information collected included demographic data, co-morbidities, clinical status, anthropometric measures, medication use, laboratory results, and electrocardiogram (ECG) findings. Additional information was obtained using the Duke Activity Status Index (DASI), along with patient-reported symptoms such as fatigue or dyspnoea suggestive of cardiac dysfunction.

### HF staging definition

Study participants were classified into HF stages according to the 2022 American College of Cardiology/American Heart Association guidelines.^16^ Individuals at risk of HF (Stage A) were defined as those with no structural or functional cardiac abnormalities but who had one or more risk factors, including hypertension, T2DM, or obesity. Stage B (pre-HF) was defined as asymptomatic individuals with a history of myocardial infarction or the presence of specific echocardiographic abnormalities as defined by guideline thresholds: LVEF <50%, regional wall-motion abnormalities, left ventricular (LV) hypertrophy (LV mass index >115 g/m^2^ in men or >95 g/m^2^ in women), LV dilatation (end-diastolic volume index >74 mL/m^2^ in men or >61 mL/m^2^ in women), structural heart disease, or Grade II or III diastolic dysfunction with elevated filling pressure. Diastolic dysfunction was classified using the 2016 American Society of Echocardiography recommendations, which incorporate transmitral inflow, tissue Doppler imaging, left atrial volume index, and tricuspid regurgitation peak velocity.^17,18^

Participants who presented with symptoms such as fatigue or dyspnoea attributable to cardiac dysfunction and who could be managed in the outpatient setting were classified as having symptomatic HF (Stage C). Given the community-dwelling nature of the population, Stage D (advanced HF) was not expected to be present.

### Novice training programme, AI-POCUS quick scan protocol, and workflow

Novice biomedical and bioscience graduates underwent a structured 4-week programme comprising 1 week of theoretical instruction and 3 weeks of hands-on ECHO practice. The training focused on a simplified three-view ECHO acquisition protocol (parasternal long-axis, apical four-chamber, and apical two-chamber).

Post-training, they were deployed to the diabetes primary-care spokes to perform rapid cardiac ultrasound triage using handheld ultrasound devices (Kosmos, EchoNous), equipped with AI-guided image acquisition support and United States Food and Drug Administration (US FDA)-approved AI-powered analytical software (Us2.AI). The system enabled the capture of echocardiographic images and automated measurement of cardiac parameters, including LV dimension, LV geometry, LV end-diastolic volume index, LV endsystolic volume index, LVEF, left atrial volume index, and detection of LV hypertrophy, LV dilatation, left atrial enlargement, and LV systolic dysfunction.^17,19^

Acquired images and AI-generated reports were uploaded to a secure cloud platform and remotely reviewed and verified by experienced sonographers at the intermediary centre via telehealth. Any AI-POCUS study identified as abnormal or incomplete triggered a comprehensive TTE using a conventional system at the intermediary centre. The results were reviewed by clinicians at the intermediary, who determined if the patient is managed locally or referred to the tertiary cardiology referral centre (hub). The hub would provide advanced diagnostics and further evaluation, pharmacological initiation, complex case management, or procedural or device-based interventions. For patients not requiring tertiary referral, the intermediary provided continued pharmacological titration and outpatient follow-up.

### Outcomes

The primary outcome was the detection of previously undiagnosed HF, evaluated by the proportion of patients identified with Stage B (pre-HF) and Stage C HF. Secondary outcomes evaluated the system-level performance of the hub–intermediary–spoke model, including reduction in diagnostic burden, referral rates, and outpatient workload at the tertiary centre. Additional secondary outcomes assessed novice sonographer performance, including the proportion of image captures analysable by AI for LV and LA parameters following 4 weeks of training, the success rate (defined as complete three-view image capture within ten minutes), the overall proportion of complete three-view image captures, and mean duration of each AI-POCUS examination.

This study received ethical approval from the Malaysian Ministry of Health Research Ethics Committee (NMRR ID-23-02864-78I), and was conducted in accordance with the Declaration of Helsinki and approved by the local Institutional Review Board and ethics committees. Written informed consent was obtained from all participants.

### Statistical methods

Sample size determination followed the guidelines outlined by Bujang.^20^ The primary objective of this study was to evaluate the ability of the decentralized, community-based hub–intermediary–spoke model to detect previously undiagnosed HF in a high-risk diabetes population. Accordingly, the sample size calculation was based on estimating prevalence within a population. Given the large and often underdiagnosed diabetic population, the total population size was considered unknown. A prevalence rate of 50.0% was assumed, with a 5.0% margin of error. The use of a 50.0% prevalence estimate is recommended as it yields the maximum required sample size for a fixed margin of error. Based on these parameters, the minimum required sample size was calculated to be 384 participants. To account for an anticipated non-response rate of 20.0%, the final minimum sample size for the study was set at 480 participants.

Descriptive statistics were used to summarize participant characteristics and clinical parameters. Frequencies and percentages (%) were reported for categorical variables, while means and standard deviations (SD) were used for continuous variables. Data were also presented visually using charts, box plots, and survival plots to enhance interpretability. To compare the mean duration of AI-POCUS examinations between the initial 400 cases (representing the learning curve threshold) and subsequent cases, an independent sample *t*-test was applied. Additionally, Pearson's Chi-square test was used to assess associations between the two groups with respect to the proportion of AI-POCUS studies achieving complete three-view image capture. A *P*-value of less than 0.05 was considered statistically significant, and P-value interpretation adhered to established statistical standards.^21^ All statistical analyses were conducted using SPSS version 17.0 (SPSS Inc., Released 2008. SPSS Statistics for Windows, Version 17.0, Chicago: SPSS Inc.).

## Results

A total of 1000 patients with T2DM and no prior diagnosis of HF were screened over a seven-month period across the six diabetes primary-care clinics. Of these, 985 patients had complete data and were included in the final analysis (*Table 1*). The mean age of the cohort was 61.7 ± 11.5 years, and 55.7% were women. Prevalence of comorbidities was high, including hypertension (91.2%), dyslipidaemia (88.1%) and CKD (21.2%). Approximately one in five patients had a family history of HF. The mean DASI score was 35.72 ± 12.32, indicating mild functional limitation, with an estimated mean metabolic equivalent (MET) of 7.13 ± 1.51. Glycaemic control was suboptimal (mean HbA1c 7.92 ± 2.11%). Renal impairment (eGFR <60 mL/min/1.73m^2^) was present in 19.1% of the patients enrolled. This profile reflects a typical high-risk primary care population and provided the clinical context for downstream AI-POCUS screening.

**Table 1:** Demographics and clinical characteristics

Clinical characteristics	*N* = 985
*Demographics*	
Age, years	61.7 ± 11.5
Female gender	549 (55.7)
*Medical history*	
Hypertension	898 (91.2)
Dyslipidaemia	868 (88.1)
CKD	209 (21.2)
History of coronary artery disease	21 (2.1)
History of stroke	43 (4.4)
Family history of HF	198 (20.1)
*Anthropometric measurements and vital signs*	
BMI, kg/m^2^	27.72 ± 5.28
Systolic blood pressure, mmHg	143.0 ± 17.4
Diastolic blood pressure, mmHg	81.9 ± 10.4
*Functional capacity*	
DASI	35.72 ± 12.32
DASI-derived METs	7.13 ± 1.51
*Laboratory parameters*	
HbA1c	7.92 ± 2.11
eGFR <60 mL/min/1.73m^2^	188 (19.1)

Continuous variables were expressed as mean ± SD and categorical variables expressed as *n* (%).

BMI, body mass index; CKD, chronic kidney disease; DASI, Duke Activity Status Index; eGFR, estimated glomerular filtration rate; HbA1c, glycated haemoglobin; HF, heart failure; METs, metabolic equivalents.

### Primary outcome: detection of previously undiagnosed HF

The primary outcome was the detection of previously undiagnosed HF. Among the 985 patients included in the final analysis, 87.9% (*n* = 866) were categorized as Stage A (at risk), 11.1% (*n* = 109) as Stage B (pre-HF), and 1.0% (*n* = 10) as Stage C HF (symptomatic HF). Importantly, all Stage C cases represented previously undiagnosed HF despite no prior history of HF.

### Secondary outcome: system-level performance of the hub–intermediary–spoke model

**Figure 2:**
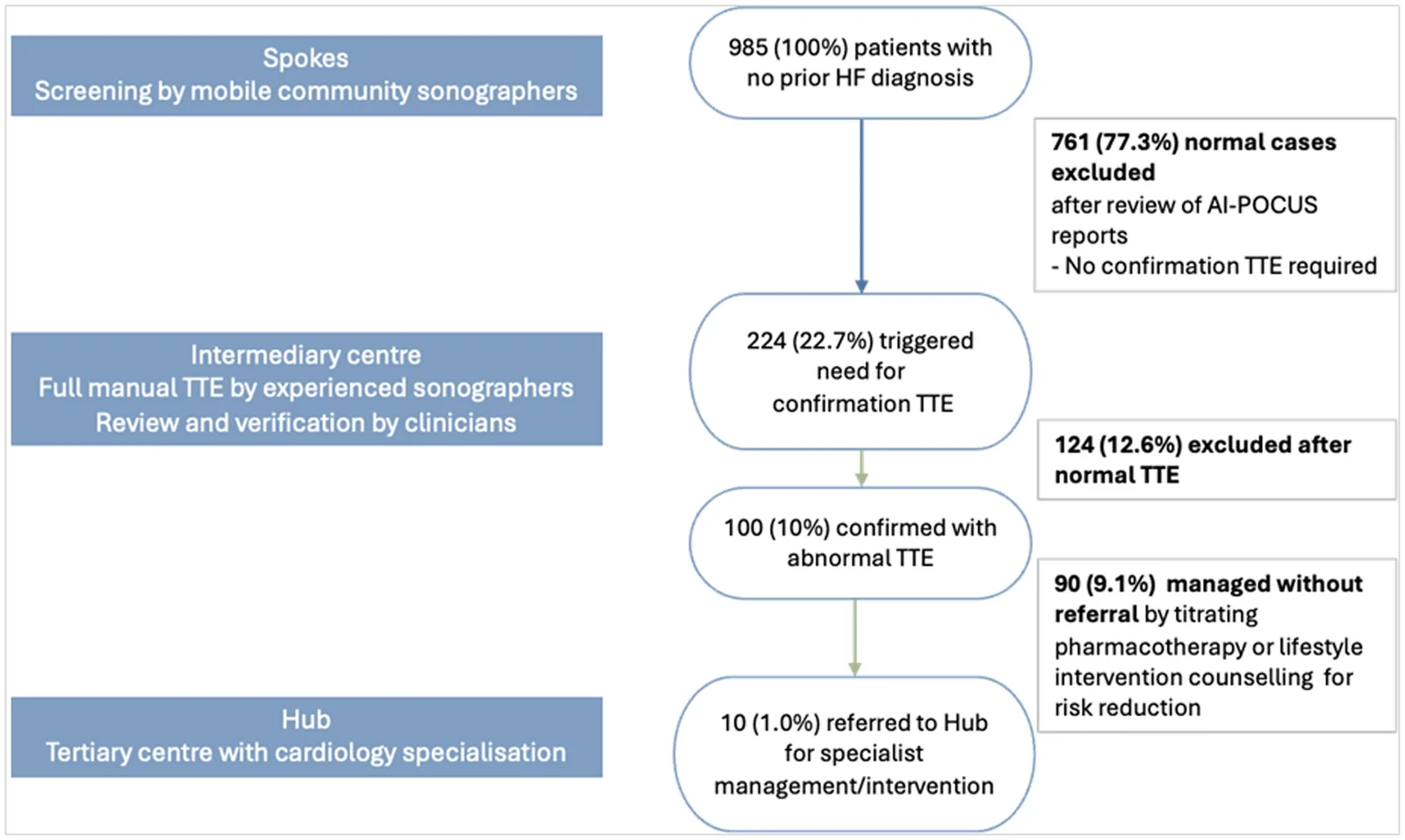
The hub-intermediary-spoke model for HF screening at diabetes primary care centres utilizing AI-POCUS performed by mobile community sonographers reduced diagnostic, referral, and outpatient management burden at the tertiary centre. AI-POCUS, artificial intelligence-powered point-of-care ultrasound; HF, heart failure; TTE, transthoracic echocardiogram.

Of the 985 patients screened at the primary-care spokes, 77.3% with normal AI-POCUS findings were ruled out after remote review without requiring further confirmatory TTE (*Figure 2*). The remaining 22.7% proceeded to intermediary-level confirmatory TTE, where a further 12.6% were excluded following normal findings. Only 100 patients (10.0%) were confirmed to have abnormal TTE findings. Among them, 9.1% were managed at the intermediary level through lifestyle modification or pharmacological optimization, while only 1.0% required escalation to the tertiary cardiology hub for specialist assessment. Overall, the hub– intermediary–spoke pathway reduced tertiary diagnostic burden by 89.9%, with most cases resolved within the spoke–intermediary loop.

### Secondary outcome: novice sonographer performance

**Figure 3:**
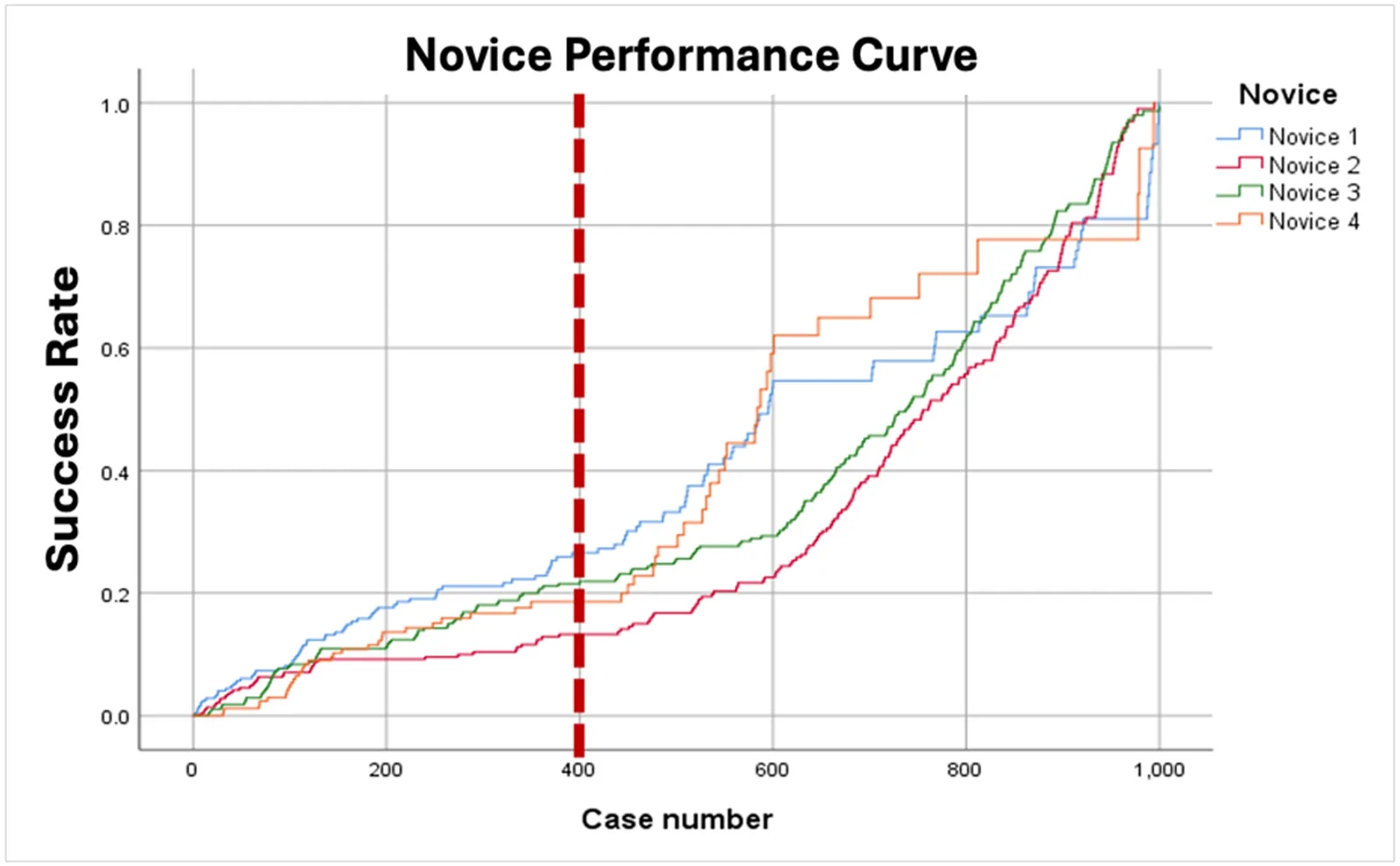
Feasibility of the hub-intermediary-spoke screening model using the success rate of completed three-ECHO views captured within ten minutes as a parameter. ECHO, echocardiogram.

Following a structured 4-week training programme, novice sonographers achieved high AI analysability across key echocardiographic parameters. Images captured by novice sonographers yielded >90% analysability by AI for key LV parameters (dimensions, hypertrophy, volumes, and ejection fraction) and >85% for left atrial volume (*Table 2*). All four novice sonographers demonstrated exponential improvement in success rate—defined as complete three-view capture within 10 min—after approximately 400 cumulative cases (*Figure 3*). The proportion of complete three-view captures increased significantly from 88.0% to 92.2% (*P* = 0.035). Efficiency also improved, with mean examination time reduced from 11.03 ± 5.28 min during the first 400 cases to 8.31 ± 4.41 min thereafter, corresponding to a mean difference of 2.31 min (95% CI: 1.52–3.11; *P* < 0.001).

**Table 2:** Percentage of analysable scans by the four mobile community sonographers after 4 weeks of training

ECHO parameter	Yield, *n* (%)
Intraventricular septal thickness in diastole	966 (98.1)
Left ventricular posterior wall thickness in diastole	953 (96.8)
Relative wall thickness	949 (96.3)
Left ventricular mass index	949 (96.3)
Left ventricular end-diastolic volume index	897 (91.1)
Left ventricular end-systolic volume index	898 (91.2)
Left ventricular ejection fraction	896 (91.1)
Left atrial volume index	874 (88.7)

ECHO, echocardiography.

## Discussion

HF remains a global health challenge, particularly in low- and middle-income countries (LMICs) where access to diagnostics is limited and patients often present late in the disease course. The Heart2Miss initiative represents a paradigm shift in HF detection, pioneering a hub-intermediary-spoke model, supported by AI-POCUS and task-shifting to novice biomedical and bioscience graduates, to enhance early diagnosis and intervention. Our findings demonstrated that this decentralized, community-based approach could expand diagnostic reach in primary care, achieve diagnostic scalability and optimize specialist resource use. Among patients with T2DM, 11.1% were identified as having Stage B (preHF) and 1.0% had previously undiagnosed Stage C HF. Trained novice community AI-POCUS sonographers captured a high proportion of analysable images, and most cases were resolved at the spoke-intermediary level, significantly reducing the diagnostic burden on tertiary referral centres.

Primary care clinics remain the foundation of community healthcare, delivering critical first-contact services for chronic disease populations, especially high-risk groups such as older adults and those with T2DM. These clinics are vital and trusted access points, particularly in LMICs.^22,23^ The success of community HF screening programmes, such as Brazil's national initiative, demonstrates the feasibility of integrating HF screening within public health frameworks and leveraging the influence of community healthcare.^24^

Despite their importance, diagnostic capacity in resource-constrained primary care settings remains limited, challenged by financial constraints, lack of ECHO systems, and healthcare workforce shortages.^25,26^ The acquisition and maintenance of conventional ECHO systems require significant capital and operational costs. Additionally, the extensive training typically required for dedicated sonographers (6 months to 2 years) poses a further barrier to integrating ECHO services. In Sarawak, for example, there were 29.2 nurses per 10 000 population in 2021,^27^ only slightly above the figure reported by the World Health Organization's 2020 *State of the World's Nursing* report.^28^ It was reported that LMICs typically have fewer than 20 nurses per 10 000, far below the 80 nurses per 10 000 in high-income countries. Additionally, over half of the surveyed nations reported an insufficient nursing workforce for primary care delivery.

To address these challenges, we adopted a scalable and pragmatic hub-intermediary-spoke model suited for resource-limited environments. This model extended ECHO diagnostics from tertiary hospitals into the broader community by leveraging the primary care's local reach. Employing portable POCUS devices and AI-supported analyses, minimized infrastructure costs while enabling community healthcare providers with minimal prior experience to conduct focused cardiac ultrasound (FoCUS) assessments.^29^

The decision to employ a three-view image capture protocol was based on established FoCUS principles. Current international guidelines recognize that comprehensive TTE, which typically involves 30 to 40 views, is not always feasible or necessary in community or acute care settings. Instead, FoCUS protocols, which employ three to five simplified views, have been validated for the rapid assessment of LV function and volume status, especially in screening and triage contexts.^30,31^ By utilizing a streamlined three-view protocol, our study reduced the time required for each screening examination compared with full TTE assessments, increasing throughput and feasibility in primary care settings. This approach also aligned with the training and skill set achievable by novice sonographers within a 4-week training period.

The intentional exclusion of Doppler and tissue Doppler imaging parameters in Heart2Miss represented a carefully considered trade-off justified by three factors. First, the triage-first objectives of community screening. In primary care, the priority is not definitive HF phenotyping, which requires specialist evaluation, but efficient identification of abnormalities warranting referral while excluding normal cases. Our strategy proved critical to reducing unnecessary tertiary diagnostic burden by 89.9%, as most patients only needed confirmation of absence of disease.

Second, novice-friendly acquisition. Simplified scanning protocols allow novice operators to reliably perform ECHO examinations after limited training while still capturing essential parameters for initial triage. In our study, >85% analysable scans were achieved using the three-view protocol mastered in 4 weeks vs. months needed for comprehensive exam protocols.

Third, US FDA-cleared AI validation with converging external evidence. The protocol leverages a US FDA-cleared automated workflow with robust validation across diverse populations (Us2.AI)^19,32^ demonstrating similar accuracy to manual measurements by expert sonographers. The studies revealed that automated measurements by AI not only achieved expert-level agreement but also outperformed human inter-reader variability for key echocardiographic parameters. For real-world applicability in LVEF quantification,^33^ the clinical validation of handheld POCUS showed high sensitivity and specificity for detecting LV dysfunction (LVEF <50%), with excellent test–retest reliability. A multicentre study assessing FoCUS with AI-supported LVEF assessment demonstrated novice and experienced users achieving highly reproducible LVEF estimations compared with formal TTE [intraclass correlation coefficient 0.904 and area under the curve (AUC) 0.98 for detecting LVEF <50%].^34^

The evidence was expanded further by demonstrating that after minimal training, nurses and nurse-assistants could capture analysable echocardiographic images for AI-supported LV hypertrophy detection.^35^ The model complemented the findings of the CUMIN study^36^ showing the feasibility of rapid training for nurses, thereby strengthening the evidence that AI-supported focused protocols can extend diagnostics into low-resource settings. These findings were further supported by the PANES-HF study, which demonstrated strong novice performance after 2 weeks of training (AUC 0.88) with short scan times.^37^ Training duration across studies ranged from one day (CUMIN)^36^ to 2 weeks (PANES-HF)^37^ and 4 weeks (Heart2Miss), reflecting differences in operator background, intended clinical application, and image acquisition complexity. Despite these variations, all three studies demonstrated that AI-supported echocardiography can be successfully deployed by non-expert operators after relatively brief training periods.

Our focused three-view protocol, optimized for speed and novice-friendliness, expanded access to community cardiac assessment without compromising triage value. Novice biomedical and bioscience graduates achieved >90% analysability for LV structure and function, and >85% for left atrial volume. Initial mean scan time was 11 min and reduced further as operators gained experience. Only 22.7% of cases required confirmatory TTE after expert review. Beyond HF detection, AI-POCUS applications continue to expand demonstrating accurate AI-based identification of hypertrophic and transthyretin amyloid cardiomyopathies from >90 000 POCUS videos.^38^

In resource-constrained settings, tertiary centres often lack the capacity to support primary care clinics adequately. The limitations of the classic hub-and-spoke model, which relies on a central hub providing specialized services to primary care spokes, are well recognized. Such systems often produce bottlenecks at the hub level due to excessive referral volumes delaying care, undermining confidence in the spokes and compromising system efficiency.^39^

Our hub-intermediary-spoke model addresses these challenges by incorporating an additional tier between the spokes and hub. This intermediary tier served multiple roles, including delivering standardized training for novice mobile sonographers, remotely verifying AI analyses, performing confirmatory TTE when indicated, and managing Stage B HF through risk-factor optimization and pharmacological titration before discharging patients back to primary care. With this stepwise cascade, most cases were resolved within the spoke—intermediary loop, successfully reducing the tertiary cardiology hub diagnostic burden. Only 1.0% of the screened population required escalation to the tertiary cardiology hub, while 9.1% were safely managed at intermediary centres via lifestyle or pharmacological intervention.

The hub-intermediary-spoke cascade not only preserved tertiary capacity for patients with advanced disease but also exemplified how AI-POCUS, telehealth, and structured workflows can redistribute diagnostic tasks across levels of care. The implications extend beyond HF with the application of similar cascades to other non-communicable diseases where community-based early detection is critical. From a policy perspective, the intermediary tier does not increase the overall burden but rather functions as a diagnostic filter and care coordinator. It strengthens the spokes through feedback and telehealth support while reinforcing tertiary capacity.

The scalability of this approach has been demonstrated not only in our study but also in similar models applied elsewhere in LMICs.^23,40^ The sustainability of such models, however, depends on workforce flexibility, a requirement addressed in our initiative through synergistic task-shifting. In settings where healthcare workforce shortages persist, an oversupply of biomedical and bioscience graduates leads to underemployment and a mismatch between education and available career pathways.^41,42^ Hence, task-shifting to science graduates presents a dual opportunity. By providing targeted training and upskilling, these graduates can be repurposed as mobile cardiac screeners, expanding diagnostic access without necessitating costly workforce expansion.

### Strengths and limitations

The Heart2Miss study demonstrated the feasibility of the hub-intermediary-spoke model. Our science graduates achieved over 85% analysable cardiac ultrasound triage scans after completing training in weeks, which traditional sonography training would require months or years to accomplish. In seven months, novice sonographers screened 1000 high-risk patients, eliminating local community ECHO backlogs. Importantly, this workforce innovation did not compromise quality. The significant reduction in unnecessary tertiary referrals demonstrated that non-medical personnel, when supported by AI verification and intermediary oversight, could perform effective triage.

Findings from the CUMIN study^36^ reinforce this. In that study, five novice nurses trained for one day successfully performed home-based AI-POCUS in Tunisia, achieving interpretable LVEF in 80% and left atrial volume index (LAVi) in 68% of patients, with 92% sensitivity for detecting cardiac dysfunction. By comparison, in the present Heart2Miss study, novice biomedical and bioscience graduates achieved analysable LV parameters in over 90% and LAVi in over 85% of cases. These complementary results demonstrate that brief training, combined with AI support, enables non-expert operators to conduct effective cardiac screening across diverse settings and populations.

This approach demonstrates how rethinking workforce structures can simultaneously close diagnostic gaps and address graduate underemployment—a dual benefit with major policy relevance. For policymakers, it offers a scalable strategy to align education outputs with population health needs, advance equity, and strengthen health system resilience.

Several considerations should be acknowledged when interpreting these findings. First, this was a single-region study, which may limit generalizability to other populations with different healthcare infrastructures or demographic profiles. Nevertheless, the core principles underpinning the model—AI-supported task-shifting, telehealth-enabled quality assurance, and a tiered hub-intermediary-spoke referral structure—may be transferable to other resource-constrained healthcare systems facing similar workforce and diagnostic access challenges. The intermediary tier may be particularly relevant in settings where tertiary centres are overstretched and primary care lacks access to specialized diagnostics, as it provides a pragmatic mechanism for centralizing training, quality assurance, confirmatory testing, and referral coordination. This enables primary care participation in cardiac screening while preserving specialist capacity for patients with greater clinical complexity.

Second, while novice sonographers demonstrated rapid skill acquisition and achieved high image analysability by AI, their performance relied on structured training, intermediary verification, and AI support. Third, the three-view FoCUS protocol, while pragmatic for triage, may not capture all abnormalities detectable by comprehensive ECHO. Balancing simplicity, efficiency and diagnostic breadth remains a key design challenge. Fourth, this study lacks prospective evaluation of the model in terms of morbidity, mortality, healthcare utilization, and economic impact. Finally, although the task-shifting approach showed promise, scalability across diverse healthcare systems will require adaptation to local workforce and resource capacities, digital infrastructure, and policy frameworks.

### Implications for clinical practice, policy changes and future research

Despite its limitations, Heart2Miss advances three levels of innovation: (i) diagnostic innovation through AI-enabled community ultrasound; (ii) workforce innovation via biomedical and bioscience graduate training; and (iii) system innovation through the hub– intermediary–spoke cascade. Together, these represent a scalable, sustainable, equity-oriented model for CV prevention in LMICs. It is recommended that future research focus on evaluating how the model performs under different levels of supervision and technological support, as well as assess long-term patient outcomes, formal health-economic outcomes, and transferability across diverse systems. As digital health and AI tools evolve, their integration into workforce and system redesign will be central to achieving population-scale early detection and prevention of HF.

## Conclusion

The Heart2Miss initiative demonstrated that a decentralized, community-based AI-POCUS screening model, configured through a hub–intermediary–spoke cascade, can effectively identify previously undiagnosed HF and substantially reduce diagnostic and referral burden at the tertiary care level. By combining portable imaging technology, AI-supported analyses, and targeted training of novice biomedical and bioscience graduates, this model extended diagnostic capacity into primary care, improved efficiency, and preserved specialist resources. Importantly, it not only closed diagnostic gaps but also addressed workforce underemployment, aligning educational outputs with health system needs, a dual benefit of major policy relevance. Broader adoption of such models could offer a pragmatic pathway for resource-limited settings to strengthen HF prevention, optimize resource utilization, and advance equity through integrated system workforce redesign.

## Data Availability

All data underlying this article are incorporated into the article.
